# A Taxonomic Framework Based on Machine Learning for Unsupervised Anomaly Detection in Healthcare Data

**DOI:** 10.3390/healthcare14203380

**Published:** 2026-10-10

**Authors:** Gelson André Schneider, Moisés Laurence de Freitas Lima Júnior, Davenilcio Luiz de Souza, Domingos Rafael Ferla Valladares, Mikael Henrique de Jesus Batista, André Luís Korzenowski

**Affiliations:** 1 Federal Institute of Education of Tocantins (IFTO), Araguaína Avenida Paraguai, Esquina com a Avenida Amazonas (Quadra 56, Lote 01), Setor Cimba, Araguaína 77824-838, TO, Brazil; 2 Federal Institute of Education of Tocantins (IFTO), Araguatins Povoado Santa Teresa, Km 05–Zona Rural, Araguatins 77950-000, TO, Brazil; moises.junior@ifto.edu.br; 3 Independent Researcher, R. Zeferino Dias, 151/118-Sarandi, Porto Alegre 91130-480, RS, Brazil; davenilcio@gmail.com; 4 Business School, Pontifical Catholic University of Rio Grande do Sul (PUC-RS), Av. Ipiranga, 6681-Prédio 50, 10º andar–Partenon, Porto Alegre 90619-900, RS, Brazil; domingos.valladares@pucrs.br; 5 School of Health Sciences (FCS), Federal University of Northern Tocantins (UFNT), Av. Dionísio Farias, 838–Loteamento Bairro de Fátima, Araguaína 77814-350, TO, Brazil; mikael.batista@ufnt.edu.br; 6 Coterflex, Rua Vereador Mário Cardoso Ferreira, 959–Distrito Industrial, Alvorada 94836-195, RS, Brazil; andre@korzenowski.com

**Keywords:** anomaly detection, taxonomic framework, unsupervised machine learning

## Abstract

**Objectives:** This study presents a generic taxonomic framework as a roadmap for users who wish to implement systems for detecting fraud and waste in health data; this roadmap outlines which unsupervised machine learning techniques can be used to detect anomalies in health data, and whether these anomalies are point, contextual, or collective. **Methods:** The research employed the Design Science Research (DSR) methodology in five stages: awareness, ideation, development, evaluation, and conclusion. The proposed taxonomic framework was designed through the implementation of the artifact, using the “Sample 1 to Inpatient Claims CMS 2008–2010 Data Entrepreneurs’ Synthetic Public Use File (DE-SynPUF)” database, which contains 66,773 records and 81 features. The dataset was applied to the K-Means, Isolation Forest (IF), and Local Outlier Factor (LOF) algorithms for point anomaly detection. **Results:** The number of anomalies detected by at least one of the algorithms was 13,966, representing (20.9%) of the dataset; K-Means alone detected 1608 (2.4%), IF alone detected 4522 (6.8%), LOF alone detected 2301 (3.4%), K-Means and IF together detected 4497 (6.7%), K-Means and LOF together detected 41 (0.1%), IF + LOF combined detected 465 (0.7%), and all three algorithms in consensus detected 532 (0.8%). **Conclusions:** Thus, the taxonomic framework proposed in this study can be viewed as a roadmap for researchers and developers seeking to understand which unsupervised technique to apply in detecting anomalies in health data, considering the type of anomaly.

## 1. Introduction

The healthcare sector is part of the service sector and is supported by various financing models. Healthcare services include hospitals, clinics, laboratories, and healthcare professionals; they can be obtained privately, through health insurance—in Brazil, the term “supplementary health insurance” is used—or fully or partially funded by the government. One example is Brazil’s Unified Health System (SUS), a government program that offers free care to the population. This system can operate through its own infrastructure or rely on the private network, which is duly reimbursed. Precisely because of the high concentration of public and private resources, the healthcare sector has become a fertile ground for fraud. The world’s major healthcare fraud cases are listed in a 2017 report by the OECD (Organization for Economic Cooperation and Development) [1].

In addition, healthcare systems are bureaucratic and complex, involving numerous rules, regulations, and stakeholders, which makes it difficult to detect fraud across the various levels of these systems [2]. In Brazil, a study published by the Institute for Supplementary Health Studies (IESS) [3], conducted by the consulting firm Ernst & Young (EY), found that fraud and waste together caused a loss of approximately R$ 34 billion for health plan and insurance providers in 2022—approximately 10% of their revenue.

Fraud and abuse in the healthcare sector take many forms. The study by [4] lists some of the most common types of health insurance fraud committed by healthcare providers: (i) submitting false invoices for services that were never even provided to the patient; (ii) submitting duplicate claims for the same individual service; (iii) misrepresenting the services provided to patients; and (iv) requesting reimbursement for covered services that, in practice, were not covered by the provider.

In addition, the study by [5] also provides a list of the most common types of fraud, which are as follows: submitting requests for unnecessary ancillary services; filing an excessive number of claims for different services that should be billed as a single service; submitting reimbursement requests for amounts greater than the value of the services provided (upcoding); and submitting an invoice for services not rendered. Moreover, types of fraud are becoming increasingly sophisticated, and distinctions between fraudulent and non-fraudulent behavior are rapidly becoming obsolete, as fraudsters develop smarter and more cautious approaches to evade audits [6].

For a long time, manual fraud audit techniques, such as discovery sampling, were used to detect fraud [7]. However, to increase the effectiveness and speed of detection, computerized and automated fraud detection systems—which were initially limited—have been developed and refined over the years. Over time, they have become more complex and have integrated a wide range of data mining methods involving statistical, mathematical, and machine learning techniques to extract and identify useful information, thereby forming decision support systems and intelligent systems for the effective detection of fraud [5].

One of the techniques used to detect fraud and waste in healthcare is anomaly detection, which refers to the problem of identifying patterns in data that do not conform to expected behavior. These non-conforming patterns are generally referred to as anomalies, outliers, discordant observations, exceptions, aberrations, surprises, peculiarities, or contaminants.

Of these, “anomalies” and “outliers” are the two terms most commonly used in the context of detecting non-conforming patterns, sometimes interchangeably [8]. Anomalies can also be categorized into three types: point, contextual, and collective [8]. Therefore, fraud can be viewed as an anomaly detection problem [9]; thus, one approach for a system to detect fraud and waste in healthcare could be the use of anomaly detection techniques. Anomaly detection is also applied to various other scenarios, such as intrusion detection in computer networks, credit card fraud detection, auto and home insurance fraud detection, and the detection of faults in industrial equipment, among other applications.

In anomaly detection, algorithms are commonly used that fall into two categories. The first is supervised algorithms, which aim to classify whether a given situation is fraudulent or not based on labeled data; however, in most cases of fraud, the data is not known or labeled, and even when it is, there is very little of it, causing a class imbalance that can bias the algorithms. Another category of algorithms consists of unsupervised algorithms. These algorithms discover patterns or cluster data without the need for labels, making them a potential solution for detecting anomalies and, consequently, fraud and waste in health data.

Unsupervised methods can also be classified as probabilistic models, linear models, and proximity-based models, which are further subdivided into cluster-based, distance-based, and proximity-based models. The nature of the attributes/features will determine which anomaly detection model will be applied [8]; that is, the choice of model requires a good understanding of the data, as well as the type of target: point, contextual, or collective.

Finally, anomaly detection in healthcare can be part of a larger ecosystem: healthcare fraud and waste detection alert systems. These systems can process large amounts of data to analyze and issue alerts regarding the presence of fraud and waste in healthcare systems. However, these systems are prone to failure; due to the nature of the problems, they have a low accuracy rate and generate false alarms [10].

Despite significant research on detecting fraud and waste in healthcare, there is still no clear guideline for determining which technique to apply to each type of anomaly.

Therefore, the objective of this research is to propose a taxonomic framework as a roadmap for users who wish to implement systems for detecting fraud and waste in health data. This roadmap outlines which unsupervised machine learning techniques can be used to detect anomalies (point, contextual, or collective) in health data, taking into account challenges such as dimensionality reduction, concept drift, class imbalance, and the lack of real-time detection capabilities.

## 2. Materials and Methods

This research followed the Design Science Research (DSR) method, a methodology that focuses on obtaining solutions through the creation or modification of artifacts that satisfy specified requirements and can change situations [11], with the goal of reconciling theoretical research with practice, producing scientific knowledge to be applied in organizations through the development of solutions to real-world problems—an approach that has been explored in engineering [12] and in information systems research [13].

The paper [14] describes a guide with seven guidelines/instructions that fully characterize research conducted using the Design Science Research methodology:

(i). Develop an innovative artifact;
(ii). Identify a specific problem to which the artifact can be applied;
(iii). Evaluate its usefulness;
(iv). Build the artifact based on rigorous methodologies;
(v). Develop the artifact through research;
(vi). Use research resources efficiently;
(vii). Communicate the results to stakeholders.

Finally, the authors add that the structure of the stages can be modified according to the specifications and nature of each project. All the guidelines proposed by [14] can be incorporated into the sequence of stages proposed by [15]:

1. Awareness of the problem: Problems can arise from various sources, such as industry needs or readings that lead to field discoveries by the researcher. The outcome of this phase is a proposal for new research.
2. Suggestion: A phase in which the researcher will make attempts at suggestion. At this point, creativity is essential, and each researcher will tend to arrive at different results, since different theories can be used to explain the same phenomenon.
3. Development: In this phase, the researcher will construct their artifacts, which may be a method or a model. This does not necessarily require novelty in relation to the state of the art of the subject being studied, since the innovation lies in the construction and/or application of the artifact.
4. Evaluation: The artifact is evaluated at this stage based on the criteria outlined in the proposal. Initial hypotheses are rarely fully validated; therefore, any deviations from expectations—both qualitative and quantitative—are identified and adjusted at this stage.
5. Reflection/Conclusion: Although deviations in behavior typically still occur, the efforts made to solve the problem are considered sufficient. The results should be validated and documented in such a way that they can be generalized to a specific class of problems.

**Figure 1:**
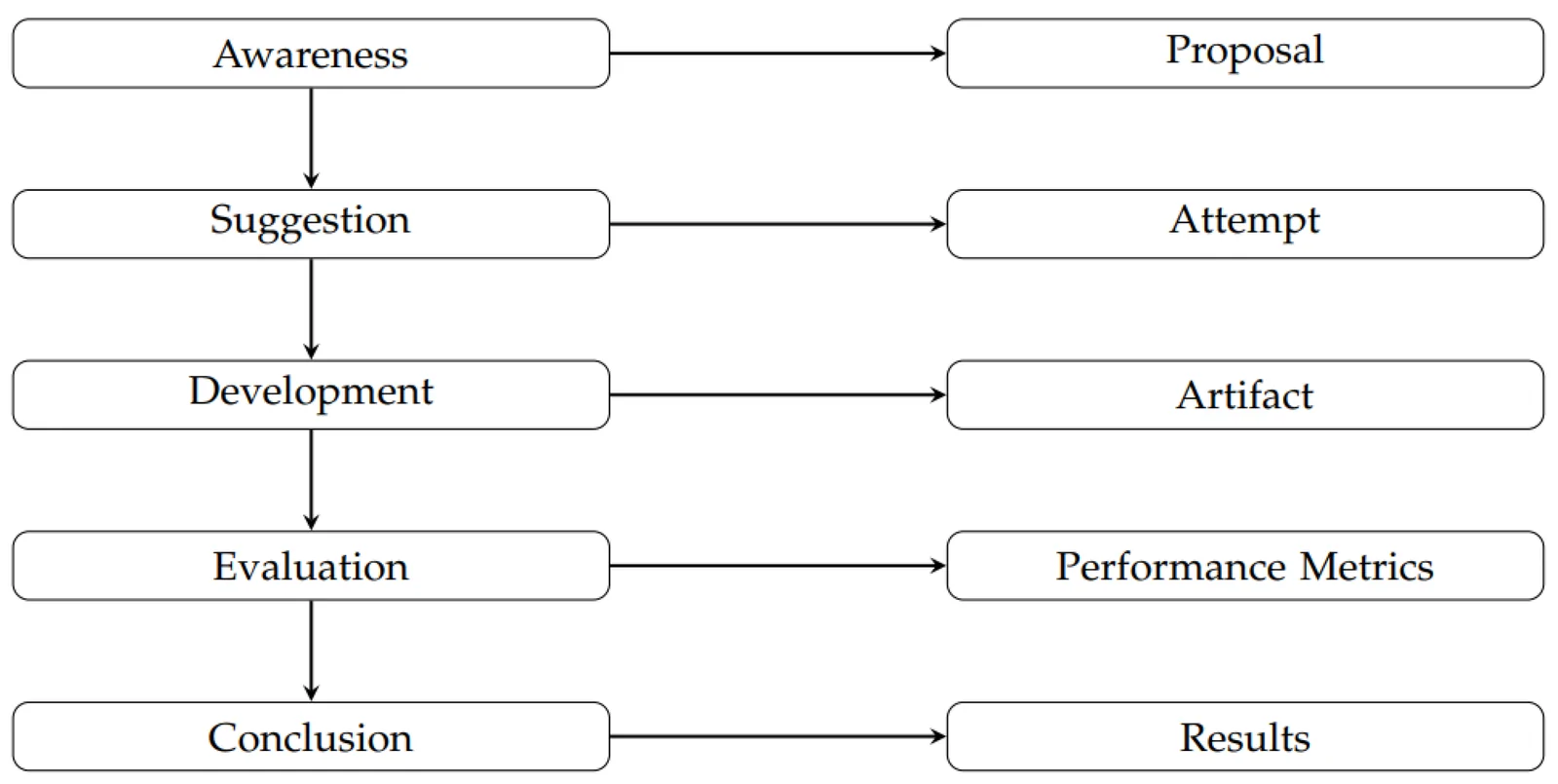
Stages of the DSR methodology, adaptad: [11].

In addition, ref. [11] notes that for each stage of the DSR methodology, there is a deliverable or output, as shown in the Figure 1.

Next, each of the five phases of the described methodology will be detailed.

### 2.1. Awareness

At this stage of the research, the goal is to raise awareness of the problem; it is necessary to identify it and conduct a systematic literature review (SLR). Identifying the problem leads to the formulation of the research hypothesis. In this specific case, the research hypothesis guiding the study is the following: “Can the detection of fraud and waste in health data using unsupervised machine learning techniques be viewed as an anomaly detection problem?”

To check our hypothesis, we conducted an SLR, through which we determined that anomalies can be classified into three types: point, contextual, and collective. We then classified the studies according to the type of anomaly and, consequently, the type of technique or algorithm applied. In Table 1, we analyze the findings of the SLR.

#### 2.1.1. Point Anomaly Detection

An anomaly is considered a point when an individual snapshot can be considered anomalous compared to the rest of the data [8]. According to the author, this anomaly is the simplest to detect and has been the focus of most research into anomaly detection, as can be seen in our current systematic review, in which six of the ten documents analyzed fall into this category, as shown in Table 1.

However, as useful as these models may be if they are the only ones used, they will not reduce the overall scale of healthcare fraud. If these models are the only ones used in the fight against healthcare fraud, it is unlikely that providers involved in fraud who take a more cautious approach will be identified [24]. On the other hand, the chance of finding false positives using this approach is very low. The field of medicine is inherently heterogeneous in terms of both health conditions and their presentation and treatment. Increasing volumes of data and rapidly changing patterns bring challenges that require innovative solutions [25].

With the large volume of data, databases often contain high dimensions, i.e., an instance with a large number of attributes, but anomaly detection methods based on Distance/Proximity/Nearest-Neighbor such as K-NN, Kth-NN, LOF and K-Means, which are commonly used in point anomaly detection, are agnostic about the geometry of the space or the heterogeneity of the data, so they do not work very well on these datasets [26].

Dimensionality reduction is a challenge addressed in this work. In our systematic review, we found only one document that brings this discussion to light: the study by [20] proposes a model for detecting point anomalies but uses a method based on factor analysis to reduce dimensionality in mixed data, i.e., continuous, discrete, and categorical attributes. The method creates a continuous, reduced-dimensional embedding that can be combined with detection algorithms for a low-dimensional continuous domain such as IF, LOF, and DBSCAN.

#### 2.1.2. Contextual Anomaly Detection

To mitigate the shortcomings of anomaly detection through point detection, we can resort to contextual anomaly detection. If a data instance is anomalous in a specific context but not in another, it is called a contextual anomaly; the notion of context is induced by the structure of the dataset and must be specified as part of the problem formulation [8]. Each data instance must have contextual attributes that are used to determine the context (or neighborhood) of that instance; for example, in time series data, time is a contextual attribute that determines the position of an instance in the entire sequence. Behavioral attributes define the non-contextual characteristics of an instance.

In this context, we classified two documents in our systematic review as contextual anomaly detection approaches. In ref. [21], the researchers carry out data mining to detect fraud/anomalies related to Diagnosis-Related Group (DGR) upcoding. For this, an unsupervised K-Means algorithm was used to identify and group locally consistent and locally similar hospitals according to the characteristics and behavior in the treatment of a specific disease. For the authors, the K-Means algorithm is more robust to variable cluster densities and high data dimensionality, and the simplest to be included in a broader algorithm that automatically selects the best subsets of resources and the best number of clusters. Finally, the proposed model was found to be promising in detecting anomalous DRG coding behavior and is easily transferable to all diseases and contexts of interest.

Already using the Medicare dataset, the study carried out by [22] demonstrates the unsupervised primitive sub-peer group analysis (PSPGA) technique based on primitive group analysis (PGA) [27]. PGA performs a local pattern analysis; the pattern of similarity in the behavior of the objects can only be identified when it is compared with the behavior of the objects in its peer group, rather than with that of the entire population. The authors’ motivation in developing PSPGA was to group providers with a similar profile using PGA and then form subgroups by analyzing the similarity in the claims of each primitive peer group. In this way, it is possible to distinguish a conceptual drift from an anomalous deviation; this distinction significantly reduces false positives, and the proposal showed better results than the K-Means, NaiveBayes, SVM, Decision tree, and KNN techniques when compared through sensitivity analyses. In future work, the authors suggest applying PGA to an online fraud detection system.

It is well known that one of the possible problems in the health system is that systematic changes occur very frequently. In this sense, contextual anomaly detection can be applied to the challenge of detecting concept changes, since this anomaly may or may not be fraud. For example, in a context where concept changes are treated as non-fraud and sudden changes as fraud, it is important to understand the distinction between them to greatly reduce false alarms in the detection process [22].

Although contextual analyses naturally have a higher false positive rate, they can identify frauds that are not well known [24]. Thus, by detecting contextual anomalies, it is possible to detect changes in concept and sudden changes, resulting in the discovery of fraud that attempts to pass for a service within the standards. However, contextual models are likely to show more false positives than methods based on finding extreme values (point), which increases costs for the health system [21]. Despite advances, it is very difficult to quantify the number of fraudulent cases that go undetected. In this sense, the two aforementioned studies seek to present solutions for identifying the challenge of concept drift.

#### 2.1.3. Collective Anomaly Detection

If a set of related data instances is anomalous with respect to the rest of the dataset, it is called a collective anomaly. The individual data instances in a collective anomaly may not be anomalies on their own, but their occurrence together with the set is anomalous [8]. In this context, we have classified two documents in our systematic review as being collective anomaly detection approaches:

(i) The task of sequential pattern mining or association rule mining, which aims to find associations between data instances, is thus a technique for detecting collective anomalies. In the study by [10], sequential pattern mining is used to identify and group courses of cancer treatments using radiotherapy that may be comparable. The method seeks to find patterns within treatments, calculate the costs of possible additional claims or unusual upcoding, and rank providers based on possible recoverable costs. According to the authors, the method was able to identify anomalous claims as well as the patterns in which they were anomalous. This work addressed the challenge of concept drift.
(ii) To analyze drug prescriptions for the automatic detection of atypical prescriptions, i.e., normal and abnormal doses of each drug regarding dosage and frequency based on prescription history data, Ref. [23] developed an unsupervised method called Density–Distance–Centrality (DDC). The method is based on graph centrality and is calculated using the PageRank algorithm so that higher scores correspond to more frequent or normal prescriptions and lower scores are anomalies, i.e., possible errors in the prescribing dose and/or frequency. Overdoses or underdoses are probably prescriptions whose centrality score is below an average centrality index for each drug. According to the authors, the method was able to quickly create a distribution pattern to detect anomalous prescriptions.

We must understand that a point anomaly detection or collective anomaly detection problem can be transformed into a contextual anomaly detection problem by incorporating context information. The techniques used to detect collective anomalies are very different from those used to detect point and contextual anomalies [8]. We can see this in the example of the contextual anomaly detection carried out by [21] using the K-Means clustering technique, a technique that is commonly used to detect point anomalies.

#### 2.1.4. Challenges

In addition to the type of anomaly, some studies identified the challenges faced in detecting anomalies in health data. Furthermore, we sought to understand how these identified challenges are currently being addressed. Thus, we identified the challenges of conceptual drift and dimensionality reduction, and related them to point, contextual, and collective anomalies.

Dimensionality reduction was addressed in a study on point anomalies, in which the technique can significantly improve the algorithms; however, we did not find any feature engineering and/or data preprocessing techniques, an important step for the replicability of studies, especially with real data. This is related to the type of study, as the studies normally use small, preprocessed datasets to test the algorithms.

Concept drift was found in three documents, two of which were related to the detection of contextual anomalies and one to a collective anomaly. Advances in the techniques show that contextual and collective methods tend to reduce false positives, a problem caused by the dynamic nature of health data, i.e., concept drift. On the other hand, the detection of anomalies in real time was not mentioned in any of the studies, which is still an open gap.

In our literature review, we did not find any studies that used a framework structure to specify which technique to apply for anomaly detection based on the type of anomaly.

To raise awareness, an SLR was conducted; the methodology and results of this review were published in the proceedings of the 25th Brazilian Symposium on Computing Applied to Health (SBCAS 2025), held in Porto Alegre, Rio Grande do Sul, from 9 to 13 June 2025, under the title “Challenges and Innovations in Healthcare Fraud and Waste Detection Systems: A Systematic Review and Proposed Framework” [28].

### 2.2. Suggestion

Based on the SLR, as shown in Table 1, insights were obtained to answer the central question of this thesis: Can we classify unsupervised machine learning techniques by type of anomaly (point, contextual, and collective) in health data to develop a generic taxonomic framework that can be replicated for other health datasets, given the challenges encountered?

**Figure 2:**
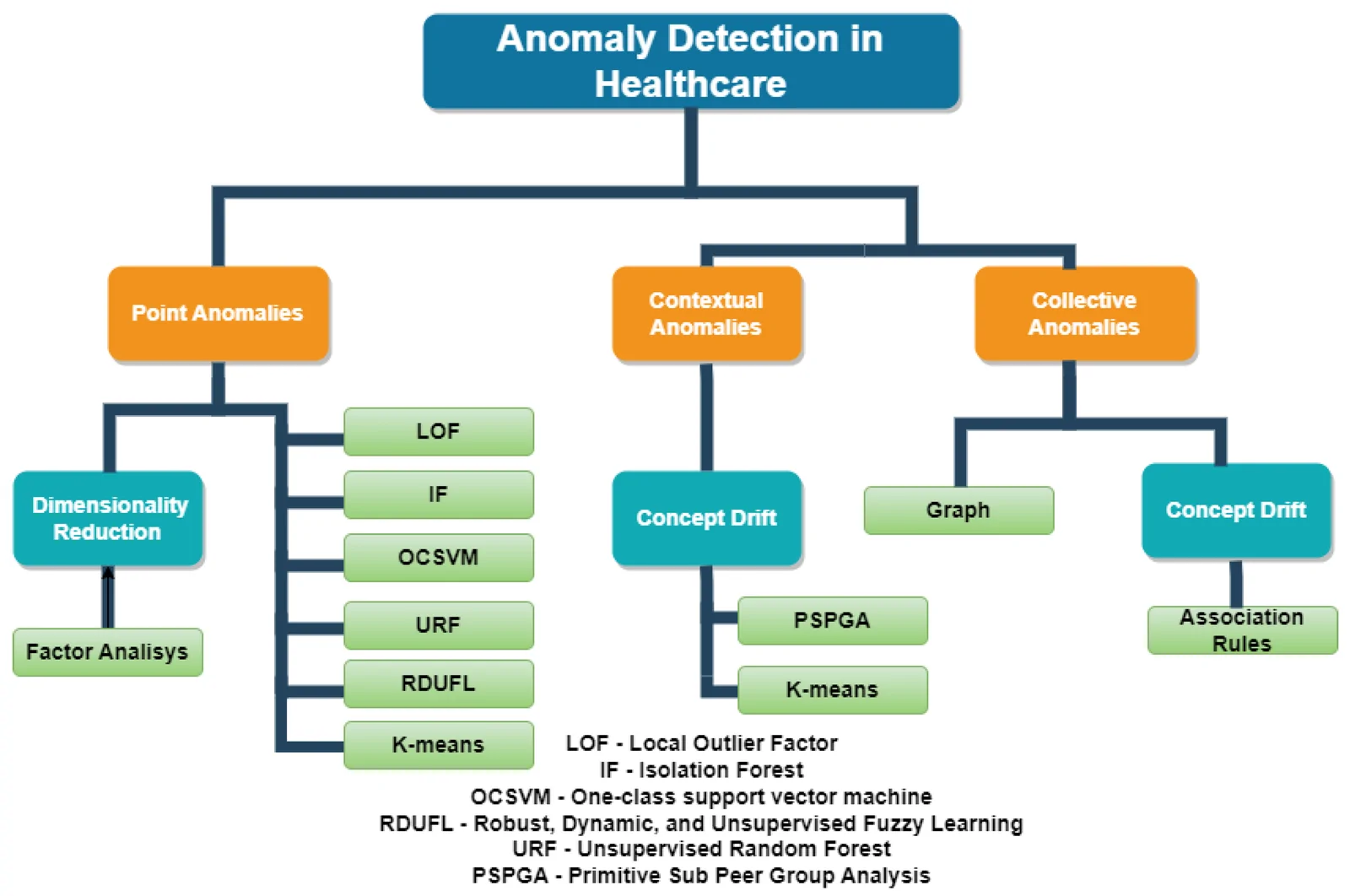
Proposed taxonomic framework.

In response to this question, Figure 2 presents the proposed taxonomic framework that classifies unsupervised machine learning techniques by anomaly type (point, contextual, and collective).

Thus, we developed our proposed taxonomic framework for classifying unsupervised machine learning techniques by type of anomaly (point, contextual, and collective); each branch covers the type of anomaly and the techniques that can be applied to that type of anomaly. For example, in the branch of point anomalies, techniques such as LOF, IF, OCSVM, URF, RDUFL, and factor analysis can be applied for dimensionality reduction.

We can see that a technique may fall under more than one category, such as K-Means, which can be used for both point and contextual anomaly detection, since, as we have already discussed, a point anomaly detection or collective anomaly detection problem can be transformed into a contextual anomaly detection problem by incorporating context information. We can see this in the example of the contextual anomaly detection carried out by [21] using the K-Means clustering technique, a technique that is commonly used to detect point anomalies.

To organize the techniques used in each type of anomaly detection and thereby assist researchers and developers, we propose the taxonomic framework presented here.

Following the DSR methodology, the development and evaluation phases are described in the results section below.

## 3. Results

In the results phase, the remaining steps of the DSR methodology are presented.

### 3.1. Development

To analyze the feasibility of our taxonomic framework, we conducted a study during the development phase to detect anomalies in the category of point anomalies using unsupervised algorithms, such as LOF [29], IF [30], and K-Means [31]. We have chosen to analyze, for now, only three algorithms that fall under the category of point anomalies; all three were selected based on the nature of their anomaly detection: (i) LOF operates in the local context, identifying points that are anomalous relative to their immediate neighborhood; (ii) in the global context, the algorithm isolates points that are, in general, rare and distinct from the entire dataset; and (iii) K-Means, which is a clustering algorithm. Its objective is to partition the data into groups of similar points. The implementation of other algorithms, as well as the categories of contextual and collective anomaly detection, will be addressed in future work.

For the development phase, the artifact branch was implemented based on the proposed taxonomic framework; for this purpose, the following database was used: DE-synPUF (https://www.cms.gov/data-research/statistics-trends-and-reports/medicare-claims-synthetic-public-use-files/cms-2008-2010-data-entrepreneurs-synthetic-public-use-file-de-synpuf (accessed on 2 March 2026)) (the Centers for Medicare and Medicaid Services (CMS) Linkable 2008–2010 Medicare Data Entrepreneurs’ Synthetic Public Use File).

This is a publicly accessible database made available for the development of technological solutions and academic research. The database contains twenty random subsample files with data on inpatients, outpatients, and medication administration, each subsample containing 5% of synthesized data randomly extracted from Medicare beneficiaries from 2008 to 2010 and their health insurance claims from 2008 to 2010.

In order to protect beneficiaries’ privacy and significantly reduce the risk of re-identification, a significant amount of interdependence and covariance among the variables was altered during the data anonymization process. This process significantly reduces the analytical utility of the dataset for drawing reliable inferences about the actual population of Medicare beneficiaries (i.e., the univariate statistics and regression coefficients produced using DE-SynPUF will be biased). Although synthesized, the dataset preserves the detailed data structure and metadata for the main variables at both the beneficiary level and the claim level.

Although there are no restrictions on the use of the entire dataset, this study utilized the dataset of inpatients from subsample 1, as studies have suggested that fraud involving inpatients may be more prevalent than fraud and waste involving outpatients [32].

Once the dataset containing subsample 1 of reimbursement claims for inpatients has been selected, each row in the dataset represents a hospital admission claim and contains 81 variables (features or attributes), which can be categorized into identifiers (IDs)—for example, CLM_ID, which is the unique identifier for the claim; provider data, which includes information about hospitals, clinics, and physicians; temporal information about the event (admission, discharge, start and end of coverage during hospitalization); information on diagnoses and procedures, such as clinical codes describing the patient’s condition at admission and during the hospitalization, as well as the treatments performed; and monetary and accounting values associated with the claim, such as days of hospitalization and the beneficiary’s coinsurance amount, among other payments. For more information on the description of each *feature*, see https://www.cms.gov/files/document/de-10-codebook.pdf-0 (accessed on 2 March 2026).

After the database was set up, the following operations were performed:

- Preprocessing, to select the attributes to be used;
- Descriptive analysis of the data, summarized due to the synthetic nature of the data;
- Detection of isolated outliers using unsupervised algorithms, such as LOF [29], IF [30], and K-Means [31];
- Evaluation of the results generated by the models.

The operations were carried out using the Python language. The requirements, the database used, and the scripts can be accessed in the GitHub repository (https://github.com/gelsonschneider/AnomalyDetection (accessed on 4 October 2026)).

The computational environment, hardware specifications, and versions of Python and the relevant libraries used in the experiments are as follows: Microsoft Windows 11 Pro 64-bit operating system, AMD Ryzen 7 7700X processor (5.4 GHz max turbo, 40 MB cache, AM5 socket, 8 cores), 16 GB RAM at 5200 MHz DDR5, Python version 3.6+ as the programming language, and the following major Python libraries: matplotlib_venn, pandas, numpy, matplotlib, seaborn, and scikit-learn.

#### 3.1.1. Preprocessing

The original dataset can be found at DE-synPUF (https://www.cms.gov/data-research/statistics-trends-and-reports/medicare-claims-synthetic-public-use-files/cms-2008-2010-data-entrepreneurs-synthetic-public-use-file-de-synpuf (accessed on 2 March 2026)) (the Centers for Medicare and Medicaid Services (CMS) Linkable 2008–2010 Medicare Data Entrepreneurs’ Synthetic Public Use File). We selected the subsample 1 file: DE1_0_2008_to_2010_ Inpatient_Claims_Sample_1.csv. Each row in the file represents a summary hospital admission claim; the data covers the period from 2008 to 2010 and includes 81 attributes, with 66,773 records (as shown in Table 2 of the User Manual, which lists the counts for each subsample).

Based on the codebook, we can categorize the attributes as follows: (i) identifiers (IDs) used to link tables, but not for predictive analysis; (ii) beneficiary demographic data, specifically the foreign key DESYNpUF_ID, which links to the beneficiaries table; (iii) provider data, which consists of information about hospitals and physicians; (iv) dates and temporal information about the event (admission, discharge, etc.); (v) diagnoses and procedures—clinical codes describing the patient’s condition and the treatments performed; (vi) financial data—monetary amounts associated with the claim; and (vii) administrative/accounting data—other metrics such as length of stay and line-item details.

During the preprocessing stage, 16 attributes were selected; Table 2 provides a description of each attribute.

The attributes were selected based on an understanding of the business model—that is, according to their importance for detecting anomalies in the health insurance claims database. For example, the LOS (length of stay) attribute was created, which represents the total number of days a patient remained hospitalized, from the date of admission to the date of discharge or death; thus, very long hospital stays (>30 days) may indicate complex clinical cases or problems, while very short stays (<1 day) may indicate recording errors. In other words, extreme values often indicate anomalies.

Similarly, the TOTALCOST attribute combines the total hospitalization cost attributes into a single attribute, taking into account Medicare payments, payments from other insurance providers, and copayments; thus, very high values may indicate complex cases or fraud, while very low values may indicate underreporting.

The COST_PER_DAY attribute (average daily cost of hospitalization) was calculated by dividing the total cost by the number of days spent in the hospital.

The NUM_DIAGNOSTICS attribute counts the number of diagnoses for a patient recorded in the claim (attributes ICD9_DGNS_CD_1 through ICD9_DGNS_CD_10). This is important for detecting anomalies: patients with many diagnoses tend to be more complex; few diagnoses accompanied by many procedures may be suspicious; and extreme values may indicate coding errors. Similarly, we created NUM_PROCEDURES, which represents the total number of procedure codes recorded in the claim (attributes ICD9_PRCDR_CD_1 through ICD9_PRCDR_CD_6), and the NUM_HCPCS attribute, which represents the number of HCPCS (Healthcare Common Procedure Coding System) codes recorded, representing detailed procedures, supplies, and services.

The PROPORTION_MEDICARE attribute represents the proportion of the total cost that was paid by Medicare, indicating the program’s share of the hospitalization costs.

The EFFICIENCY attribute measures a hospital’s efficiency, showing how much each day of hospitalization costs. Lower values indicate greater efficiency; it is calculated by dividing the total cost by the length of stay (LOS). The COMPLEXITY attribute is a composite index that measures the complexity of a case by combining the number of diagnoses and procedures performed.

We created three binary attributes, FLAG_PAYMENT_ZERO, FLAG_HIGH_COST, and FLAG_LONG_STAY: a binary indicator that identifies whether the claim had zero Medicare payment; a binary indicator that identifies the 10% of cases with the highest total cost; and a binary indicator that identifies the 10% of cases with the longest length of stay.

Finally, the categorical features CLM_DRG_CD_freq, MDC_freq, and ADMTNG_ CATEGORY_freq were encoded using the frequency encoding method. They represent the proportion of occurrence of each DRG (Diagnosis-Related Group) in the dataset, where higher values indicate more common DRGs; the proportion of occurrence of each MDC (Major Diagnostic Category)—which groups DRGs by body systems (e.g., cardiovascular, respiratory, digestive); and the proportion of occurrences for each admission diagnosis category (first three digits of the ICD-9), identifying the primary reason for hospitalization.

To normalize the data before applying the unsupervised algorithms, the StandardScaler method from the Scikit-learn library was applied to the data. This method uses the normal distribution and, therefore, transforms each value in the data so that the mean of the normalized data is zero and the standard deviation is equal to one.

Remaining null values were filled with 0 or the median. For categorical attributes, we use frequency encoding. Further information can be found in the script in our GitHub repository (https://github.com/gelsonschneider/AnomalyDetection (accessed on 4 October 2026)).

#### 3.1.2. Descriptive Analysis of the Data

**Figure 3:**
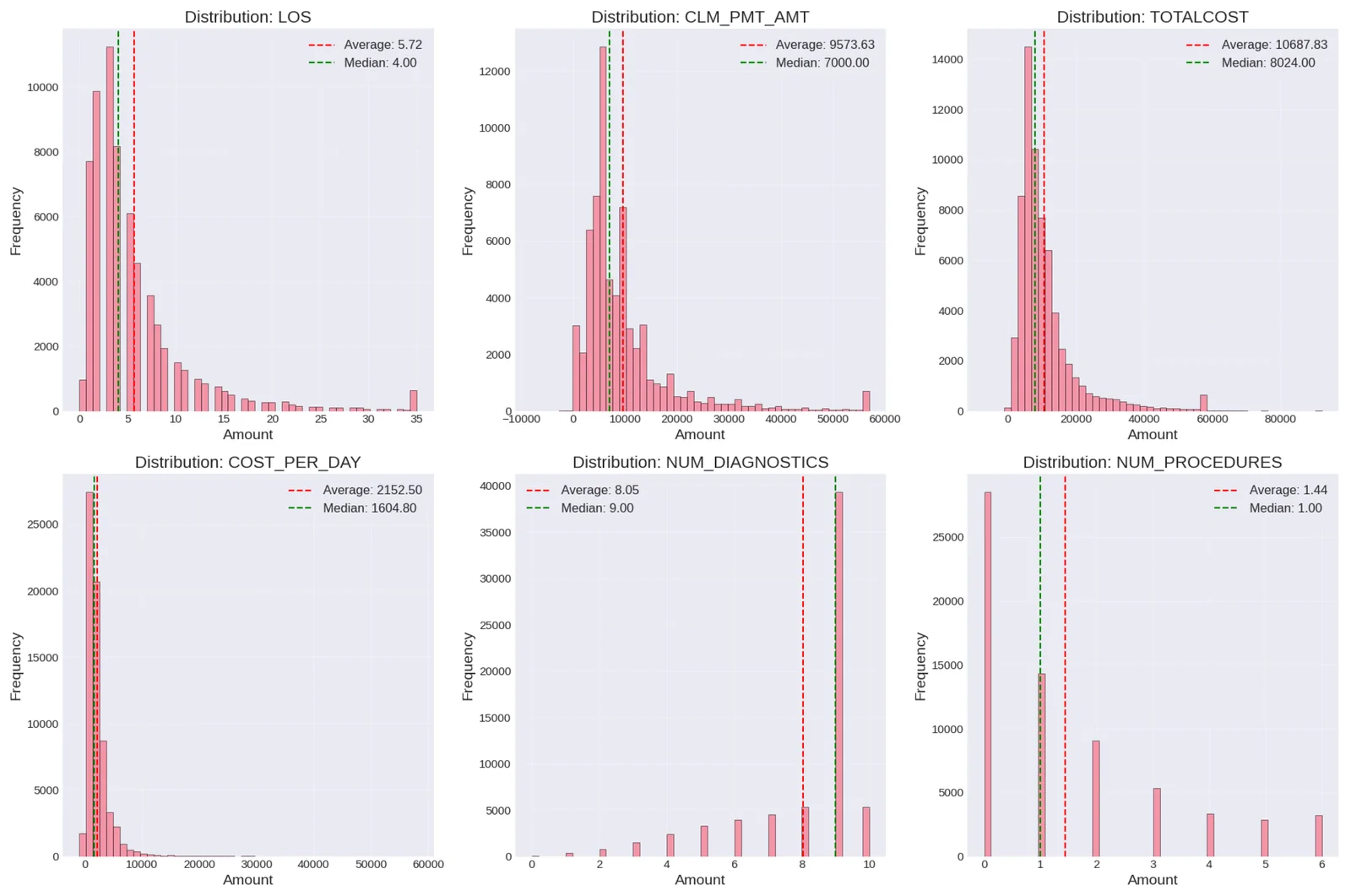
Frequency distribution of attributes.

After preprocessing, a descriptive analysis of the data was conducted to understand the nature of the data and to determine whether it aligns with the reality of the business model. To this end, the frequency distribution of certain attributes was first analyzed, as shown in Figure 3.

For the LOS attribute in days, we can see from the frequency distribution that there are hospitalizations lasting less than 1 day—which will likely be detected as an anomaly—as well as hospitalizations lasting more than 30 days, with an average of 5.72 days. We observe that the distribution is concentrated in the first few days of hospitalization, followed immediately by an exponential decline, resulting in a long tail. A similar phenomenon is observed in the total cost attribute, which shows a high frequency around the mean of 10,000 dollars, followed by an exponential decline.

**Figure 4:**
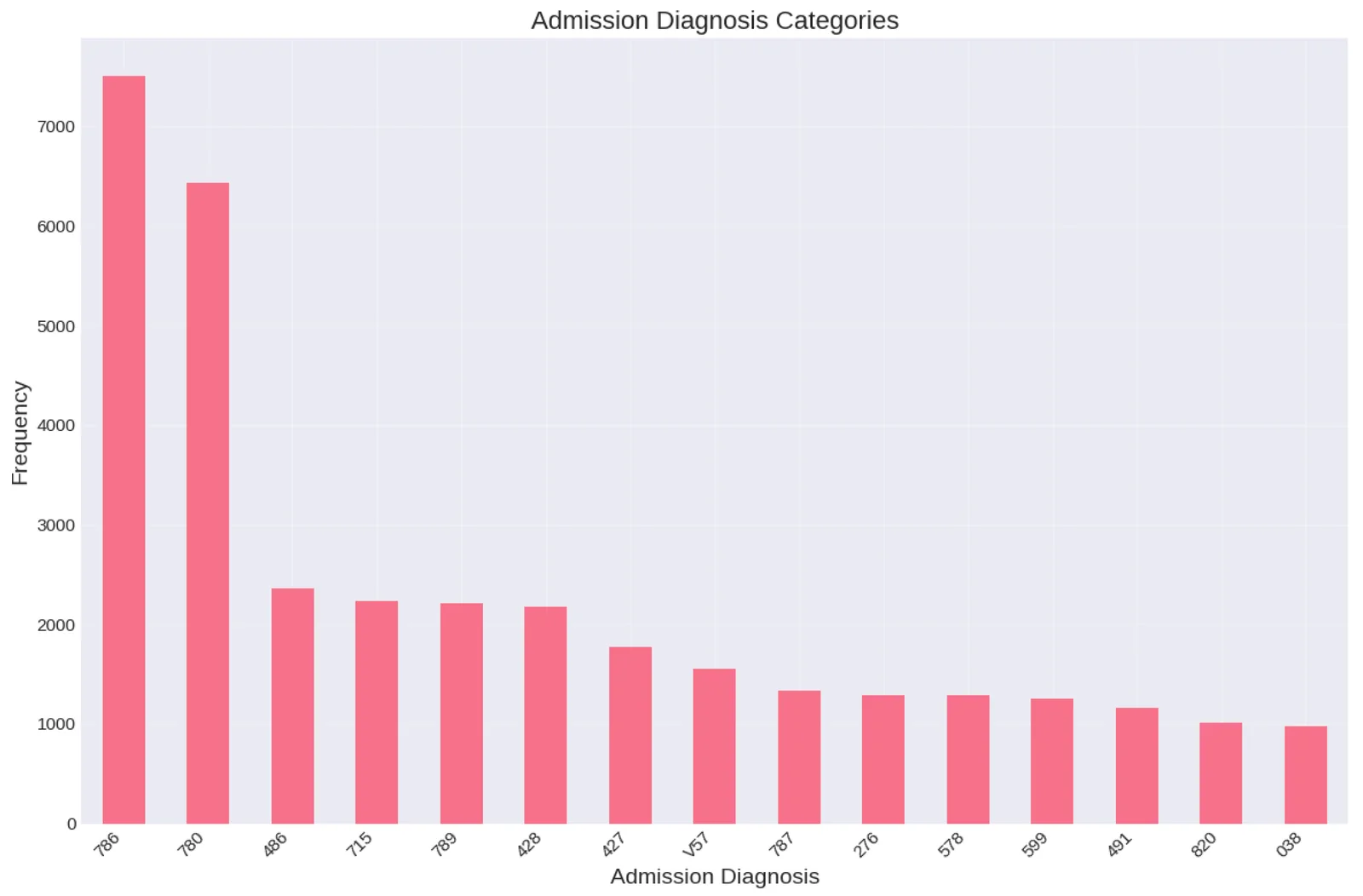
Most common diagnostic categories at admission.

Furthermore, when examining the attributes, Figure 4 analyzes the cases of admission diagnoses that are important for anomaly detection, since rare diagnoses may indicate special cases, which allows for the identification of admission patterns and is useful for defining and analyzing patient profiles.

Thus, it can be observed that most diagnoses for hospitalization are related to code 786, which in the ICD-9-CM system refers to symptoms involving the respiratory system and other chest symptoms, such as chest pain or pain when inhaling or exhaling (pleuritic). Code 780, on the other hand, is related to general symptoms such as fainting or sudden and temporary loss of consciousness, seizures (including febrile seizures), and fever of unspecified origin. There is also code 820, which refers to a specific type of fracture: a femoral neck fracture, meaning a fracture in the upper part of the thigh bone, near the hip joint.

**Figure 5:**
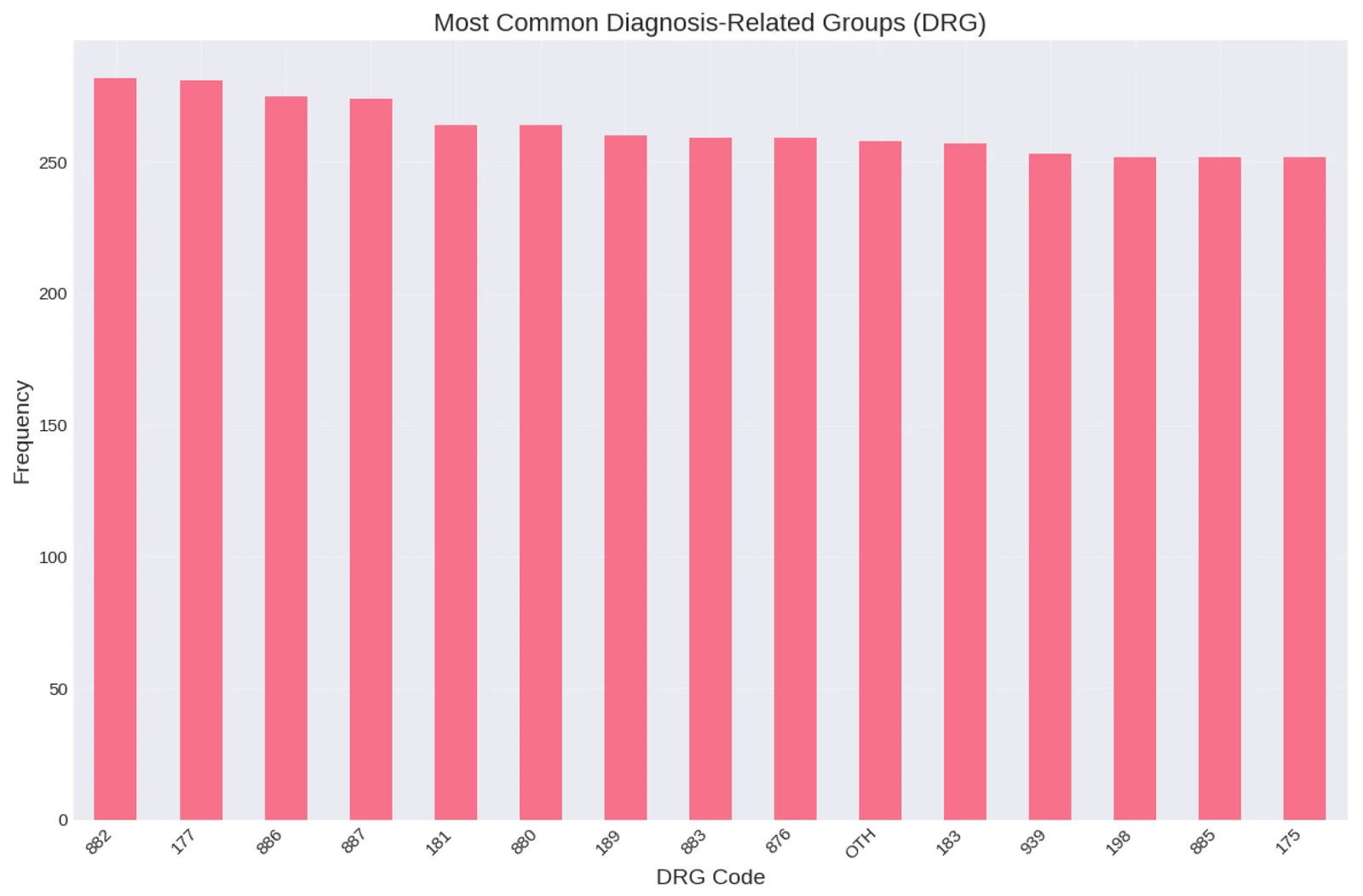
Most common DRGs.

Another important attribute for anomaly detection is the DRG (Diagnosis-Related Group), including very rare DRGs with an extremely low cost or a common DRG with characteristics typical of a rare case. Thus, Figure 5 analyzes the most frequent DRGs.

The DRG 882 code is the most common and refers to a group of psychiatric diagnoses; this DRG is classified in MDC 19 (Mental Diseases and Disorders) under the group of mental and behavioral disorders and encompasses a variety of stress-related anxiety conditions, but explicitly excludes depression, which is categorized under DRG 881.

Next is DRG 177, which relates to respiratory infections and inflammations with severe complications or comorbidities; 886—behavioral and developmental disorders; 887—other diagnoses of mental disorders; and 880—acute adjustment reaction and psychosocial dysfunction. In other words, the most common DRGs refer to diagnoses of psychiatric comorbidities.

For more information on DRG codes, visit DRG codes (https://www.cms.gov/icd10m/version372-fullcode-cms/fullcode_cms/P0002.html (accessed on 2 March 2026)).

After understanding and validating the attributes of the post-preprocessing stage described in Table 2, anomaly detection was performed using the unsupervised algorithms K-Means, IF, and LOF. The next section describes this stage and its results.

#### 3.1.3. Anomaly Detection

Using the DSR methodology during the suggestion phase, a proposal for a taxonomic *framework* for anomaly detection is presented. As shown in Figure 2, the framework features a series of unsupervised algorithms. In the development phase, in order to validate the proposal, the point anomaly branch of the artifact was instantiated with three unsupervised algorithms: *K-Means*, *IF*, and (*LOF*).

As described in Table 3, the algorithms were first parameterized.

The models were run using the optimization parameters to determine the best performance of each algorithm on the database.

### 3.2. Evaluation

Following the DSR methodology, this session involved an evaluation of the algorithms.

**Figure 6:**
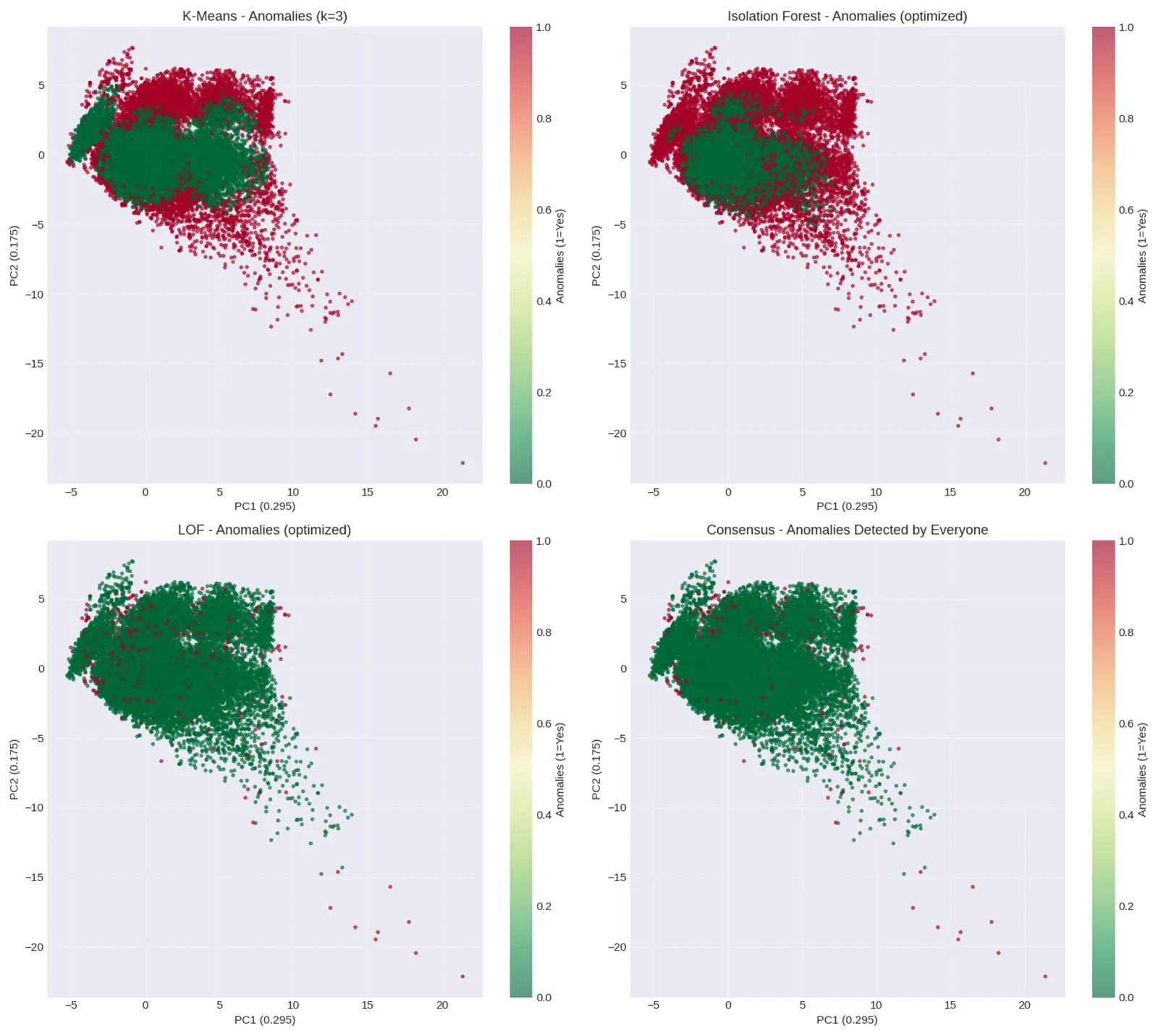
Visualization of algorithms using principal component analysis.

Figure 6 shows the database data as well as the anomalies detected by each algorithm. Based on the consensus, a principal component analysis was performed, which reduced the attributes to two principal components projected in space; the database data is shown in green, and the data considered anomalous is shown in red.

By observing the behavior of each K-Means algorithm, we can see that non-anomalous data points are grouped around the clusters. In contrast, the tree-based IF algorithm considers a data point to be anomalous based on how easy it is to isolate it relative to the extremes, while LOF considers the relative local density.

We analyzed the performance of each algorithm. IF detected 10,016 rows as anomalous, representing 15%, followed by K-Means and LOF with 10% and 5%, respectively. An important piece of data to analyze is the degree of consensus—that is, the 532 anomalies (0.8%) detected by all three algorithms, which are considered here to be the most reliable for further investigation.

Furthermore, the anomalies detected by at least one algorithm totaled 13,966 (20.9%); those detected only by K-Means totaled 1608 (2.4%); those detected only by IF totaled 4522 (6.8%); those detected only by LOF totaled 2301 (3.4%); those detected by K-Means + IF combined totaled 4497 (6.7%); those detected by K-Means + LOF combined totaled 41 (0.1%); those detected by IF + LOF combined totaled 465 (0.7%); and those detected by all three algorithms combined totaled 532 (0.8%).

We consider the 532 consensus cases to be a ranked list for manual review (audit), not confirmed anomalies. The consensus reflects agreement among methods with different inductive biases (global distance, isolation depth, local density), which may increase the efficiency of prioritization but does not constitute independent validation.

It is understood that there is little overlap among the models, indicating that they capture different types of anomalies or are sensitive to different patterns in the data.

**Figure 7:**
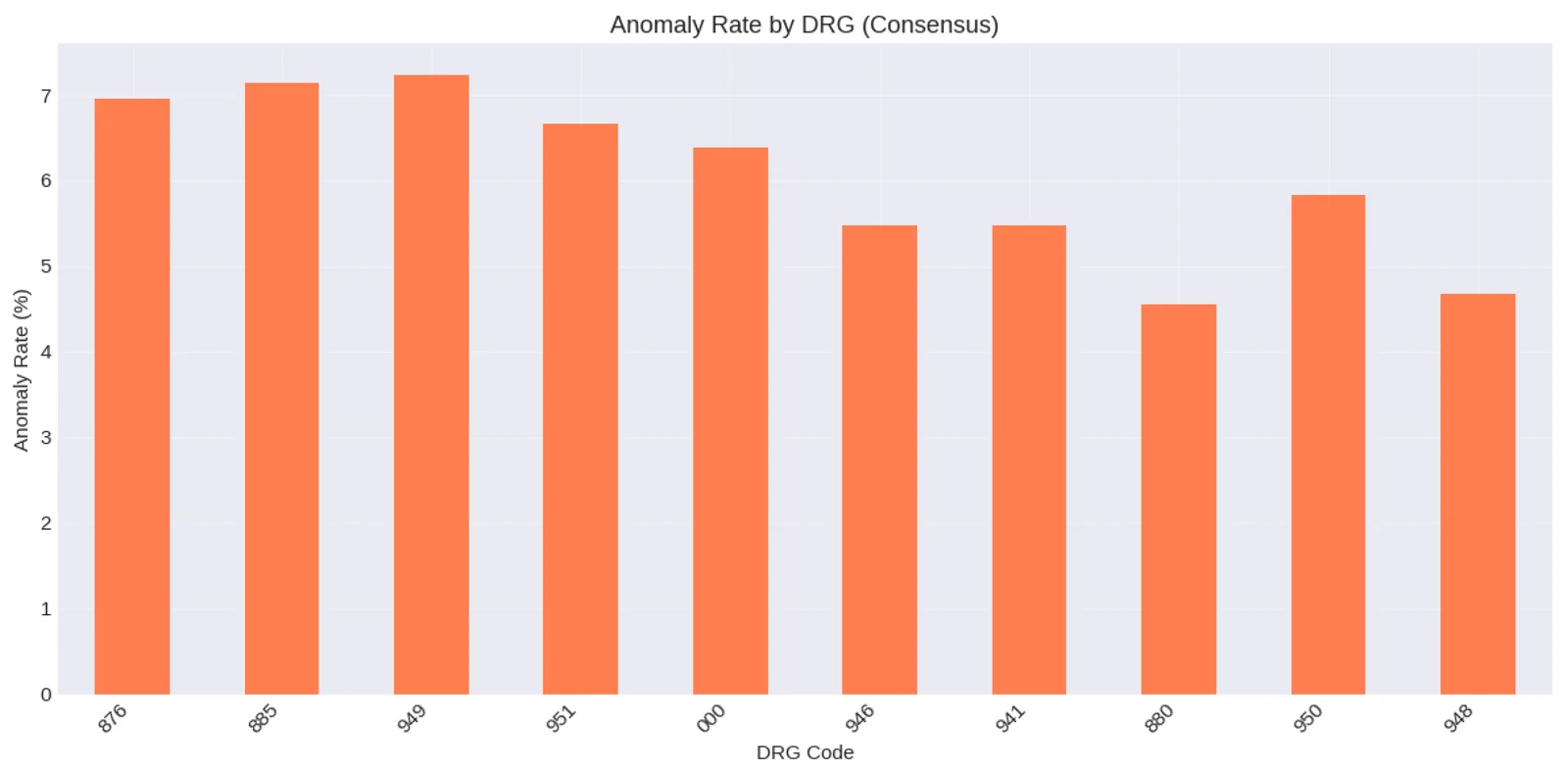
DRG anomaly rate.

By analyzing the consensus data—where anomalies are detected jointly by the three algorithms—Figure 7 shows the anomaly detection rate by diagnostic group.

Highlighted below is the ranking of the top four DRGs with the highest anomaly rates (consensus): (i) DRG 876: 6.9% anomalies; this refers to (O.R. Procedure with Principal Diagnoses of Mental Illness), a CMS group that classifies hospital admissions for patients with a primary psychiatric diagnosis who, during their hospitalization, undergo an operating room (O.R.) procedure.

(ii) DRG 885: 7.1% of cases (Psychoses); this is a CMS diagnostic group that classifies hospital admissions with a primary diagnosis of a psychotic disorder; it is one of the most common and financially significant groups within the Mental Disorders and Diseases category (MDC 19).

(iii) DRG 949: 7.2% of anomalies (Aftercare with CC/MCC); this is a CMS (Medicare Severity Diagnosis-Related Group) diagnostic group that classifies hospital admissions for follow-up care, where the patient has a Comorbidity or Complication (CC) or a Major Comorbidity or Complication (MCC).

(iv) DRG 951: 6.6% of anomalies (Other Factors Influencing Health Status); this is a CMS diagnostic group that classifies hospital admissions whose primary diagnosis is a code from the list of “Other Factors Influencing Health Status” within the category of Factors Influencing Health Status and Other Contacts with Health Services (MDC 23).

Table 4 and Table 5 analyze the cases considered the most anomalous. The most anomalous data points were selected based on the highest anomaly score—that is, the highest consensus anomaly scores. These cases arise for different reasons, the main ones being the following:

(i) Anomalies due to low cost rather than high cost, contrary to initial expectations: None of the most anomalous cases was flagged with the *FLAG* HIGH COST. The anomalies are predominantly low-cost or negative-cost, suggesting problems with data entry or external coverage.
(ii) HCPCS code count = 0 in all cases: The complete absence of HCPCS codes in the 10 most anomalous cases is a strong indicator that procedures were not properly recorded, which may explain the low costs.
(iii) Consistency pattern: high complexity + low cost: The cases with the highest complexity (9–10) and the highest number of diagnoses (9) are precisely those with the lowest costs, creating a pattern of inconsistency that the models successfully detected.
(iv) Extreme Medicare proportions: Values of 0.000 or 1.000 in MEDICARE_PROPORTION are more common in anomalous cases, indicating that cases with 100% funding from a single source or 0% funding warrant special attention.
(v) Negative payments suggest chargebacks or posting errors. Zero payments are associated with 80% of anomalous cases.

## 4. Discussion

To validate the branch of the taxonomic framework artifact, efforts were focused on detecting point anomalies; to this end, the K-Means, IF, and LOF algorithms were used. An anomaly is considered a point anomaly when a single instance can be considered anomalous relative to the rest of the data; this type of anomaly is the simplest to detect and has been the focus of most research on anomaly detection [8].

Consistent results were obtained for the chosen dataset, similar to those reported in [16], which analyzed the LOF, k-Nearest Neighbors (with five neighbors),Autoencoders, IF, and Unsupervised Random Forest to identify fraudulent providers in the Medicare Part B dataset. LOF performed best, followed by IF and Unsupervised Random Forest.

In [17], the study aimed to develop a method for detecting prescriptions with excessive (overdose) or insufficient (subdose) doses using unsupervised machine learning algorithms: OCSVM (One-Class Support Vector Machine), LOF, IF, and Robust Covariance. For the dataset in question, OCSVM achieved the best results, followed by LOF.

As useful as these models may be, if they are the only ones used, they will not reduce the overall scale of healthcare fraud. Furthermore, if these models are the only ones used in the fight against healthcare fraud, it is unlikely that providers involved in fraud who adopt a more cautious approach will be identified [24]. On the other hand, the likelihood of encountering false positives using this approach is very low. The field of medicine is inherently heterogeneous in terms of health conditions, their presentation, and treatment; thus, the increasing volume of data and rapid changes in patterns present challenges that require innovative solutions [25].

This study has limitations due to the nature of the dataset: (i) no ground truth was available; (ii) the synthetic nature of DE-SynPUF further limits the applicability of the findings to real Medicare data; (iii) the consensus should be treated as a triage heuristic, not as evidence of fraud; and (iv) any operational use would require validation by domain experts or against audited cases.

DE-SynPUF is entirely synthetic, and none of the beneficiaries correspond to actual Medicare beneficiaries. The synthesis process deliberately alters the interdependence and covariance among variables, which limits the analytical utility of the data for drawing inferences about the actual Medicare population.

The study uses only inpatient claims, and the findings may not be generalizable to outpatient claims, carrier claims, or prescription event claims (PDE), which have different structures and patterns of anomalies.

The analysis is limited to one of the twenty available subsamples, and the results may vary across subsamples due to the stochastic nature of the data generation process.

Consequently, the framework and the patterns identified should be interpreted as a methodological demonstration—that is, a roadmap for applying unsupervised anomaly detection to health insurance claims—rather than empirical findings regarding fraud or waste in the Medicare program.

## 5. Conclusions

This article proposed and validated a branch of the generic taxonomic framework to assist researchers and developers in selecting unsupervised machine learning techniques applied to anomaly detection in health data, classifying them according to the type of anomaly: point, contextual, or collective.

The research followed the Design Science Research (DSR) methodology, progressing through the stages of awareness, ideation, development, evaluation, and conclusion. In the development phase, the framework was implemented using the synthetic DE-SynPUF database, which contains 66,773 records and 81 attributes, and was tested with three unsupervised algorithms: K-Means, IF, and LOF.

The results showed the following:

- In total, 20.9% of the records were identified as anomalies by at least one of the algorithms;
- Only 0.8% (532 records) were detected by all three methods, representing cases prioritized for further investigation.

The algorithms showed little overlap with one another, suggesting that each is sensitive to different patterns of anomalies, which reinforces the importance of a combined approach.

Analysis of the most anomalous cases revealed high clinical complexities associated with very low or negative costs and payments that were either zero or fully covered by Medicare, suggesting the models’ usefulness in identifying potential anomalies.

We acknowledge that, because the data used are synthetic, they may not fully reflect the complexity and variability of real-world data; we addressed this same issue in our SLR, in which the reviewed studies rely on small or synthetic datasets, as is the case in this study. In this regard, we recommend future research to validate the framework using real-world databases, as well as to explore the detection of contextual and collective anomalies, thereby expanding the scope of the proposed framework.

As future work, we propose validating the complete structure of the taxonomic framework using real-world data to detect point, contextual, and collective anomalies using all the algorithms proposed within the framework, since in this study only the “point anomalies” branch was partially implemented using three algorithms (K-Means, IF, and LOF) on a synthetic hospital admission dataset. Thus, the contextual and collective branches remain conceptual and untested.

Finally, the taxonomic framework proposed here demonstrates the feasibility of a structure to guide the selection of unsupervised anomaly detection techniques for health data.

## Figures and Tables

**Table 1:** Summary of related work: detection techniques and open challenges.

Study	Techniques	Challenge
Point anomalies		
Bauder et al. [16]	LOF, IF, URF, AE, KNN	–
Bhaskar et al. [4]	IF, K-Means, LOF	–
Point anomalies		
Nagata et al. [17]	OCSVM, IF, LOF, RC	–
Putina et al. [18]	RHF, IF, LOF, HBOS, OCSVM, KNN	–
Dik et al. [19]	RDUFL, FCM, NC	–
Davidow et al. [20]	FAMDAD, IF, SPAD	Dimensionality reduction
Contextual anomalies		
Massi et al. [21]	**K-Means**	Concept drift
Collective anomalies		
Settipalli et al. [22]	PSPGA	Concept drift
Kemp et al. [10]	Association rules	Concept drift
dos Santos et al. [23]	DDC, OCSVM, IF, LOF, GM, RC	–

Note: IF: Isolation Forest; LOF: Local Outlier Factor; OCSVM: One-Class SVM; AE: Autoencoder; RC: Robust Covariance; GM: Gaussian Mixture; RHF: Random Histogram Forest; HBOS: Histogram-Based Outlier Score; URF: Unsupervised Random Forest; FCM: Fuzzy C-Means; NC: Normalized Cut; SPAD: Symbolic Principal Component Analysis.

**Table 2:** Post-processing attributes used in the model.

Attribute	Type	Description
Clinical and cost variables		
LOS	Int	Total length of stay (days)
CLM_PMT_AMT	Float	Total amount paid by Medicare for the hospital stay
TOTALCOST	Float	Total cost of hospitalization
COST_PER_DAY	Float	Average daily cost of hospitalization
NUM_DIAGNOSTICS	Int	Number of diagnoses per hospitalization
NUM_PROCEDURES	Int	Number of procedures performed during the stay
NUM_HCPCS	Int	Number of HCPCS codes (procedures, supplies, and services)
PROPORTION_MEDICARE	Float	Fraction of total cost paid by Medicare
EFFICIENCY	Float	Cost per day of hospitalization
COMPLEXITY	Int	Case-mix complexity index
Binary flags		
FLAG_PAYMENT_ZERO	Binary	Indicates whether Medicare payment was zero
FLAG_HIGH_COST	Binary	Top 10% of cases by total cost (>p90)
FLAG_LONG_STAY	Binary	Top 10% of cases by length of stay (>p90)
*Categorical frequency encodings*		
CLM_DRG_CD_freq	Cat.	Frequency of each Diagnosis-Related Group (DRG)
MDC_freq	Cat.	Frequency of each Major Diagnostic Category (MDC), grouping DRGs by body system
ADMTNG_CATEGORY_freq	Cat.	Frequency of the admission diagnosis category

**Table 3:** Parameters of anomaly detection algorithms.

Parameter	K-Means	IF	LOF
Best Settings (Optimized)			
k n_estimators n_neighbors	k = 3 (Optimized)	n_estimators = 50 max_samples = 0.8	n_neighbors = 30
Contamination	-	15%	5%
Metric	Euclidean	-	Euclidean
Optimized			
Method	Elbow + Silhouette	Grid Search	Grid Search
Range	k = 2 to 10	contamination = [0.05, 0.10, 0.15]	contamination = [0.05, 0.10, 0.15]
		n_estimators = [50, 100, 200]	n_neighbors = [10, 20, 30, 50]
		max_samples = [‘auto’, 0.5, 0.8]	metric = [euclidean, manhattan, minkowski]
Detection			
Threshold	Percentile 90	-	-
Criterion	Distance > Threshold	labels == −1	labels == −1
Score	center distance	decision_function	negative_outlier_factor

**Table 4:** Clinical characteristics of the most anomalous cases.

Attribute	C1	C2	C3	C4	C5	C6	C7	C8	C9	C10
# Number of diagnoses	9	9	9	7	9	9	9	10	9	9
# Number of procedures	0	1	2	1	4	1	0	2	1	0
# HCPCS codes	0	0	0	0	0	0	0	0	0	0
DRG code	884	867	894	537	949	597	884	020	868	439
MDC code	88	86	89	53	94	59	88	02	86	43
Primary diagnosis	296	518	303	715	715	682	296	723	038	573
High-cost flag	0	0	0	0	0	0	0	0	0	0

*Notes:* C1–C10: cases 1–10. HCPCS: Healthcare Common Procedure Coding System codes; DRG: Diagnosis-Related Group; MDC: Major Diagnostic Category. Case descriptions: C1: long-term stay with very low daily cost; C2: negative financial values; C3: zero payment with recorded cost; C4: zero payment with category “OTH”; C5: LS + ZP + extremely low daily cost; C6: ZP with high complexity; C7: same pattern as C1; C8: ZP with maximum complexity; C9: negative values with 100% Medicare coverage; C10: most extreme case (LS + ZP + low cost).

**Table 5:** General characteristics of the most anomalous cases.

Attribute	C1	C2	C3	C4	C5	C6	C7	C8	C9	C10
LOS (days)	35	9	3	4	21	6	35	3	10	20
Total cost (USD)	8024	−976	1024	1024	1100	1100	8024	1068	−1976	1024
Payment (USD)	8000	−2000	0	0	0	0	8000	0	−3000	0
Medicare prop.	0.872	1.000	0.000	0.000	0.000	0.000	0.872	0.000	1.000	0.000
Efficiency (USD/day)	222.88	−97.60	256.00	204.80	50.00	157.14	222.88	267.00	−179.63	48.76
Complexity	9	10	4	6	9	9	9	10	9	9
ZP flag	0	0	1	1	1	1	0	1	0	1
LS flag	1	0	0	0	1	0	1	0	0	1

*Notes:* C1–C10: cases 1–10. LOS: length of stay; ZP: zero-payment flag; LS: long-stay flag. Monetary values are in USD (two decimals); proportions use three decimals. Case descriptions: C1: long-term stay with very low daily cost; C2: negative financial values; C3: zero payment with recorded cost; C4: zero payment with category “OTH”; C5: LS + ZP + extremely low daily cost; C6: ZP with high complexity; C7: same pattern as C1; C8: ZP with maximum complexity; C9: negative values with 100% Medicare coverage; C10: most extreme case (LS + ZP + low cost).

## Data Availability

The raw data can be found at DE-synPUF (https://www.cms.gov/data-research/statistics-trends-and-reports/medicare-claims-synthetic-public-use-files/cms-2008-2010-data-entrepreneurs-synthetic-public-use-file-de-synpuf (accessed on 2 March 2026)) (the Centers for Medicare and Medicaid Services (CMS) Linkable 2008–2010 Medicare Data Entrepreneurs’ Synthetic Public Use File). The processed data and source code can be found at Github repository (https://github.com/gelsonschneider/AnomalyDetection (accessed on 4 October 2026)).

## Abbreviations

The following abbreviations are used in this manuscript:

DRG	Diagnosis-Related Groups
IF	Isolation Forest
LOF	Local Outlier Factor
LOS	Length of Stay
SLR	Systematic Literature Review
